# The effect of aging on the frequency, phenotype and cytokine production of human blood CD4 + CXCR5 + T follicular helper cells: comparison of aged and young subjects

**DOI:** 10.1186/1742-4933-11-12

**Published:** 2014-08-23

**Authors:** Maohua Zhou, Ruqiong Zou, Huiquan Gan, Zhimei Liang, Fujun Li, Ting Lin, Yanfei Luo, Xiaoming Cai, Fang He, Erxia Shen

**Affiliations:** 1 Department of Pathology and Laboratory Medicine, Guangdong General Hospital, Guangdong Academy of Medical Sciences, Guangzhou 510080, China; 2 Department of Pathogenic Biology and Immunology, Guangzhou Hoffmann Institute of Immunology, School of Basic Sciences, Guangzhou Medical University, Guangzhou 510182, China; 3 Department of Obstetrics and Gynecology, The Third Affiliated Hospital of Guangzhou Medical University, Guangzhou 510150, China

**Keywords:** Tfh cells, Aging, IL-21

## Abstract

**Background:**

T cell-dependent B-cell responses decline with age, indicating declined cognate helper activity of aged CD4 + T cells for B cells. However, the mechanisms remain unclear. T follicular helper (Tfh) cells, a novel T helper subset, play an essential role in helping B cells differentiation into long-lived plasma cells in germinal center (GC) or short-lived plasma cells. In the present study, we proposed that there might existe changes of proportion, phenotype or cytokine production of blood Tfh cells in healthy elderly individuals compared with healthy young individuals.

**Results:**

The results showed that frequencies of aged blood CXCR5 + CD4 + Tfh cells increased compared with young subjects. Both aged and young blood CXCR5 + CD4 + Tfh cells constitutively expressed CD45RO, CCR7 and CD28, and few of these cells expressed CD69 or HLA-DR, which indicated that they were resting memory cells. There was no significant difference of IL-21 frequency production by aged blood CXCR5 + CD4 + Tfh determined by FACS compared with young individuals, however, aged PBMCs produced significantly higher levels of IL-21 evaluated by ELISA. Furthermore, there were no significant differences of percentages of IFN-γ, IL-4, IL-17 or IL-22 production by aged Tfh cells compared with their counterparts of young individuals respectively. However, frequencies of IL-17+ cells within aged CD4 + CXCR5-T cells were markedly lower than in the young individuals. Furthermore we observed different frequencies of IFN-γ, IL-17, IL-4 or IL-22 production by Tfh or by CD4 + CXCR5- cells in aged and young subjects respectively.

**Conclusions:**

Our data demonstrated that the frequencies of blood memory CXCR5 + CD4 + Tfh cells increased in the elderly population. There were similar frequencies of Th characterized cytokine production such as IL-21, IFN-γ, IL-4, IL-17 or IL-22 in aged and young Tfh cells. However, aged PBMCs produced a significantly higher amount of IL-21 compare to young subjects.

## Introduction

The immune system undergoes significant changes with aging, which likely partly contributes to increased susceptibility to infections, malignancies, inflammatory diseases, and reduced response to vaccination of elderly individuals [1,2]. Significant decline in immune function during aging is termed immunosenescence [3], and higher frequencies of inflammatory diseases of elderly people indicate that aging could be regarded as a condition of dys-regulated inflammation [4].

Age-associated alterations in immune dysfunction are found in both innate and adaptive immunity in humans. For example, age-associated changes in NK cell phenotype have been previously reported that can be responsible of functional NK cell deficiency [5,6]. DCs from elderly individuals are compromised in response to microorganisms but display increased reactivity to self-antigens associated with chronic inflammation and autoimmunity [7-9]. Most importantly, profound changes in T cell function and subsets are observed in elder individuals [10,11].

Elderly population suffers increased morbidity and mortality from infectious diseases, therefore, the elderly are highly encouraged to receive vaccinations for these infections such as influenza and pneumococcal pneumonia. However, the efficacy of vaccination reduces with aging [12-14]. The mechanisms of increased infections and declined response to vaccination partially due to declined humoral response which are mainly due to function defects in cognate helper function of CD4 + T cells and B cells. Accumulating evidence demonstrated that the genesis, numbers and function of B cell altered with aging- [15-19]. Declined cognate helper activity of aged CD4 + T cells for B cells in mice were observed [20-22]. However, this phenomenon and mechanisms are not well understoodin human beings.

A distinct subset of CD4+ helper T (Th) cells, Tfh cell (Tfh) specialize in providing cognate help to antigen-specific B cells in germinal centers (GCs). Tfh cells play a key role in the formation of GCs and differentiation of GC B cells into plasma cells and memory B cells [23,24]. In GCs, Tfh cells express master regulator transcription factor Bcl6 for their generation and surface marker CXCR5 which facilitates Tfh cells homing to B cell follicles [25,26]. IL-21 is an important effector cytokine secreted by Tfh cells and directly regulates B cell proliferation and class switching [27]. However, it is extremely challenging to obtain lymph node samples from humans to define the role of Tfh cells in normal or diseases conditions. Therefore, one study published on immunity evaluated the relationship of blood CXCR5 + CD4 + T cells with Tfh cells, and demonstrated that human blood CXCR5 + CD4 + T cells shared functional properties with Tfh cells, and appeared to represent their circulating memory compartment [28].

Therefore, in this study we determined whether there were alterations in aged Tfh cells by detecting blood CXCR5 + CD4 + T cells which represented Tfh cells in blood. And we observed CD69 and HLA-DR expression on CXCR5 + CD4 + Tfh cells from young or aged individuals respectively. Furthermore, Th characterized cytokine IL-21, IFN-γ, IL-4, IL-17 or IL-22 secreted by age or young Tfh cells were determined and compared.

## Results

### Increased proportion of peripheral blood CXCR5 + CD4 + Tfh cells from the elderly individuals

**Figure 1:**
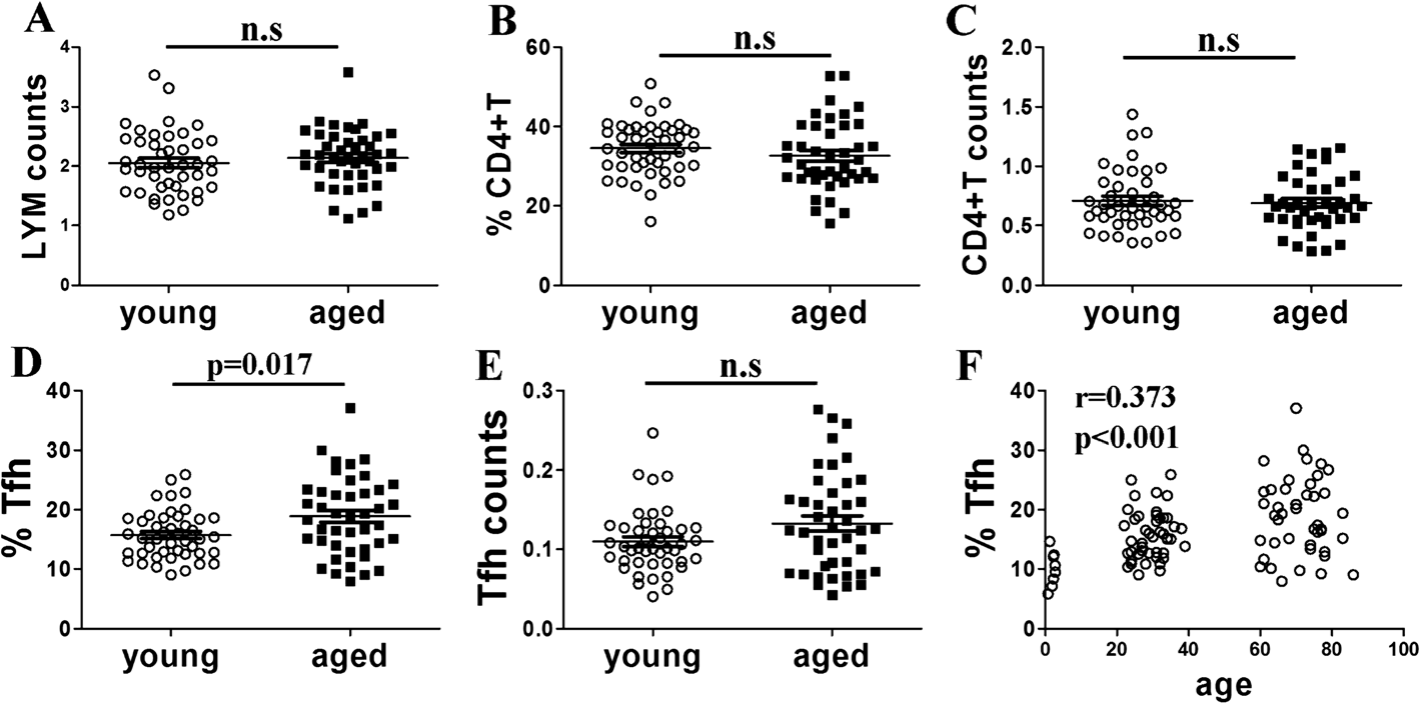
**Increased proportion of blood CXCR5 + CD4 + Tfh cells of aged subjects. A**. LYM counts: lymphocyte counts of peripheral blood (10^9^/L). **B**. Percentages of CD4 + T cells (CD3 + CD4+) in lymphocytes from young and aged groups determined by FACS. **C**. Cell numbers of CD4 + T cells in peripheral blood (10^9^/L). **D**. Frequencies of Tfh cells (CXCR5 + CD4+) in CD4 + T cells determined by FACS. **E**. The total cell counts of Tfh cells (CXCR5 + CD4+) in peripheral blood (10^9^/L). **F**. Correlation of percentages of CXCR5 + CD4 + Tfh cells with age. One open circle or filled square represented one individual. Data were expressed with line at mean with SEM.

First, we wanted to demonstrate whether cell percentages or numbers of CXCR5 + CD4 + Tfh cells changed in the elderly or not. The results showed that there was no significant difference of lymphocyte counts (Figure 1A), frequencies or cell counts of CD4 + T cells (Figure 1B, 1C) between young and aged healthy individuals. Interestingly, frequencies of CXCR5 + CD4 + Tfh cells (18.90 ± 1.004%) from elderly individuals were significantly higher compared with those from young people (15.71 ± 0.608%), and correlated with age. Furthermore, the percentages of blood Tfh cells from young children were the lower than elder individuals (Figure 1D and 1F). In addition, there was no significant difference of cell counts of CXCR5 + CD4 + Tfh cells between aged and young subjects (Figure 1E). These results demonstrated that aged blood CXCR5 + CD4 + Tfh cells proportion increased compared with young subjects.

### Memory phenotype CD45RO and CCR7, and co-stimulatory CD28 expression of aged blood CXCR5 + CD4 + Tfh cells

**Figure 2:**
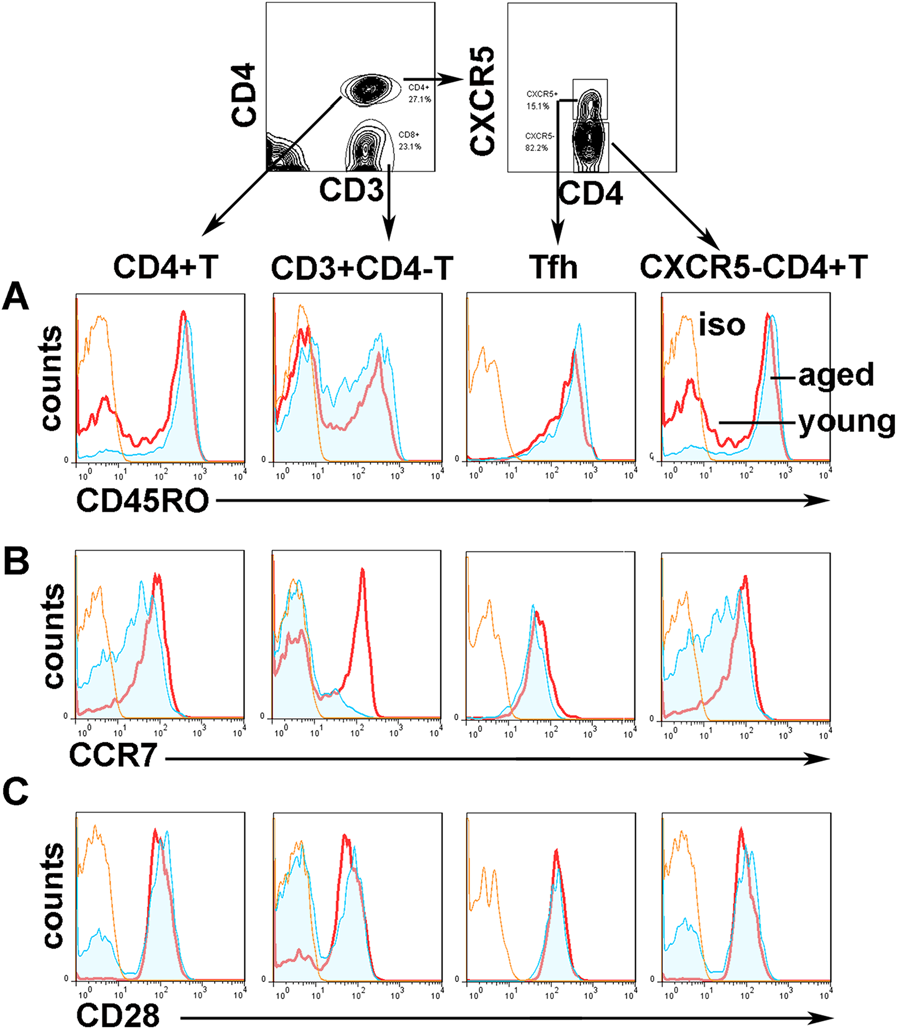
**CD45RO, CCR7, and CD28 expression on CD4 + CXCR5 + Tfh cells as well as CD4 + T, CD3 + CD4-T and CD4 + CXCR5-T cells.** CD45RO **(A)**, CCR7 **(B)** and CD28 **(C)** expression on CD4 + T, CD3 + CD4-T, CD4 + CXCR5 + Tfh as well as CD4 + CXCR5-T cells from young and aged subjects. Blood lymphocytes were first gated, and then CD4 + T (CD3 + CD4+), CD3 + CD4-T, CXCR5 + CD4 + Tfh and CXCR5-CD4+ T cells were analyzed by expression of CD45RO, CCR7 or CD28. Representative results were shown. Thin line: isotype (iso) control, heavy line: young individual (young), filled area: elderly individual (aged).

**Figure 3:**
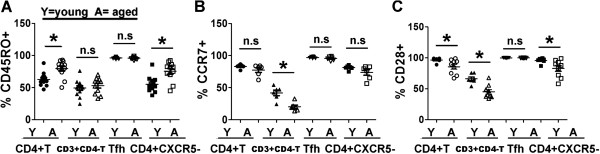
**Statistical analysis of CD45RO, CCR7 and CD28 expression on CD4 + CXCR5 + Tfh and other T subsets.** Percentages of CD45RO + **(A)** (young subjects: n = 12, aged subjects: n = 11), CCR7+ **(B)** (young subjects: n = 6, aged subjects: n = 6) and CD28 + **(C)** (young subjects: n = 7, aged subjects: n = 9) among CD4 + T, CD3 + CD4-T, CD4 + CXCR5 + Tfh and CD4 + CXCR5-T cells were shown as in histograms. Y: young individuals, A: aged individuals. *: p < 0.05, n.s: no significant difference. All data were expressed with line at mean with SEM.

We evaluated whether there were some kind of differences in the memory phenotype of CXCR5 + CD4 + Tfh cells from aged individuals compared with young individuals. First, we determined the memory phenotype CD45RO and CCR7 expression on CXCR5 + CD4 + Tfh cells using FACS. Both aged and young CXCR5 + CD4 + Tfh cells constitutively expressed CD45RO and CCR7 (Figure 2A and 2B), and there was no significantly difference between healthy aged and young individuals (Figure 3A and 3B). In addition, higher percentages of CD4 + CD45RO+, CD3 + CD4-CD45RO+, and CD4 + CXCR5-CD45RO + memory T cells were observed in aged individuals (Figure 3A). In contrast, frequencies of CCR7 positive cells among CD4+, CD3 + CD4- and CD4 + CXCR5-T cells from aged individuals decreased, and there was significant difference of percentages of CCR7 + CD3 + CD4-Tcells of elderly subjects compared with young subjects (Figure 3B).Meanwhile, we determined co-stimulatory molecule CD28 expression on blood CXCR5 + CD4 + Tfh cells using FACS. Percentages of CD28 positive cells among CD4+, CD3 + CD4- and CD4 + CXCR5-T cells from elderly individuals significantly decreased compared with their counterparts of young individuals (Figures 2C and 3C). However, there were no differences of frequencies of CD28 expression between aged and young CXCR5 + CD4 + Tfh cells. Taken together, these results indicated that Tfh cells constitutively expressed CD45RO and CCR7, and were peripheral memory T cells.

### Both aged and young blood Tfh cells are in resting state

**Figure 4:**
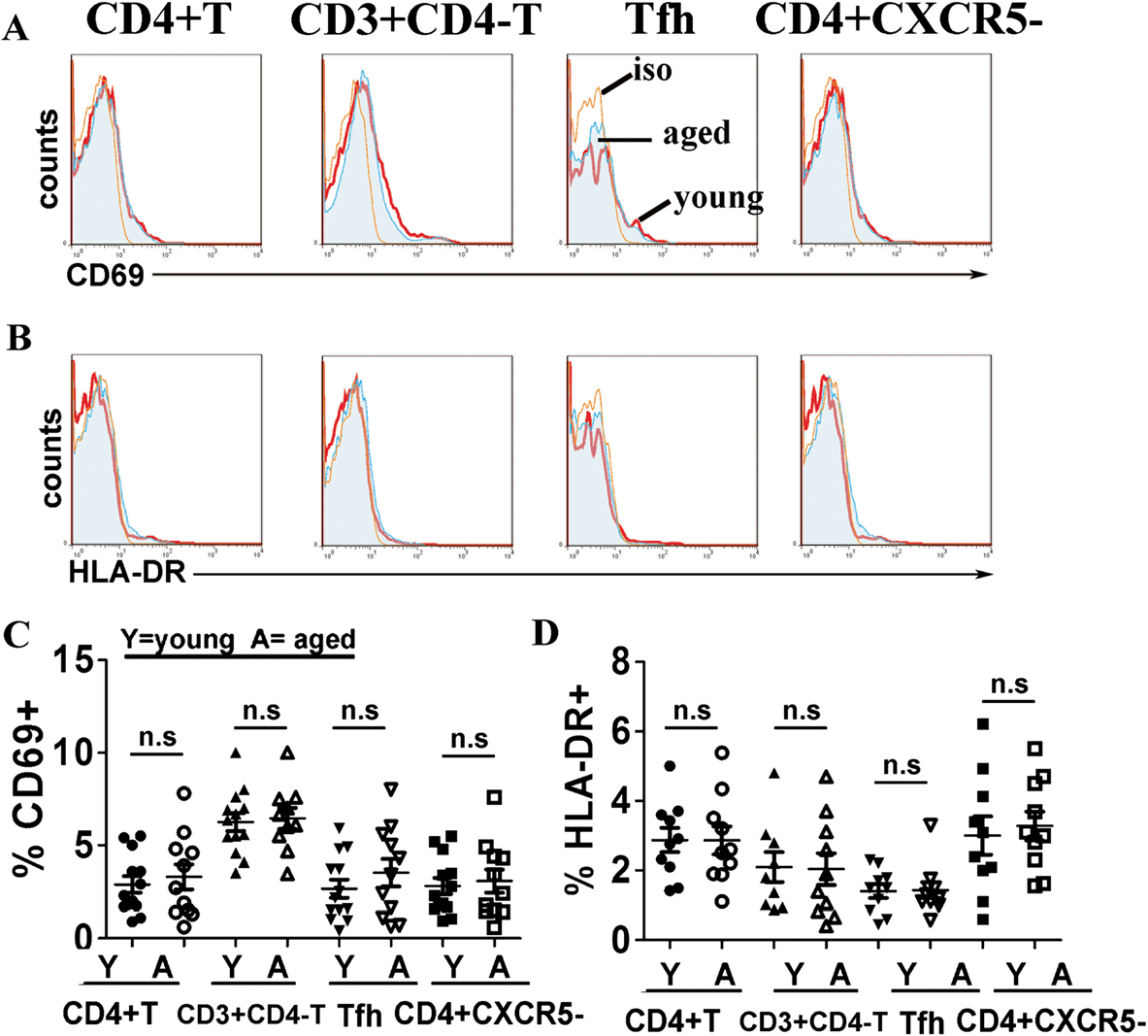
**CD69 and HLA-DR expression on young or aged CD4 + CXCR5 + Tfh cells, CD4 + T, CD3 + CD4-T and CD4 + CXCR5-T cells.** CD69 **(A)** and HLA-DR **(B)** expression on CD4 + T, CD3 + CD4-T, CD4 + CXCR5 + Tfh and CD4 + CXCR5-T cells from representative results were shown as in histograms. Thin line: isotype control (iso), heavy line: young individual (young), filled area: elderly individual (aged). Statistical results of percentages of CD69 + **(C)** (young subjects: n = 13, aged subjects: n = 11), and HLA-DR + **(D)** (young subjects: n = 10, aged subjects: n = 10) among CD4 + T, CD3 + CD4-T, CD4 + CXCR5 + Tfh and CD4 + CXCR5-T cells were shown. Y: young individuals, A: aged individuals. n.s: no significant difference. All data were expressed with line at mean with SEM.

Next, early activation marker CD69 and late activation marker HLA-DR expression on Tfh and other T subsets from young and aged individuals were evaluated by FACS. The results showed frequencies of CD69+ cells among CXCR5 + CD4 + Tfh cells, as well as CD4 + T, CD3 + CD4-T and CD4 + CXCR5-T cells from healthy elderly individuals had no significant change compared with their counterparts of healthy young individuals (Figure 4A). In addition, there were different proportions of CD69+ cells among CD4+ and CD3 + CD4-T cells both from healthy young and aged subjects. Similar with CD69 expression, few of CD4+ or CD3 + CD4-T cells subsets expressed HLA-DR, and there were no significant differences of HLA-DR expression on CXCR5 + CD4 + Tfh cells as well as on CD4 + T, CD3 + CD4-T and CD4 + CXCR5-T cells from young subjects and aged individuals (Figure 4B). Above results demonstrated that both aged and young blood CXCR5 + CD4 + Tfh cells were in resting state.

### Similar frequency of IL-21 produced by aged and young blood CXCR5 + CD4 + Tfh cells

**Figure 5:**
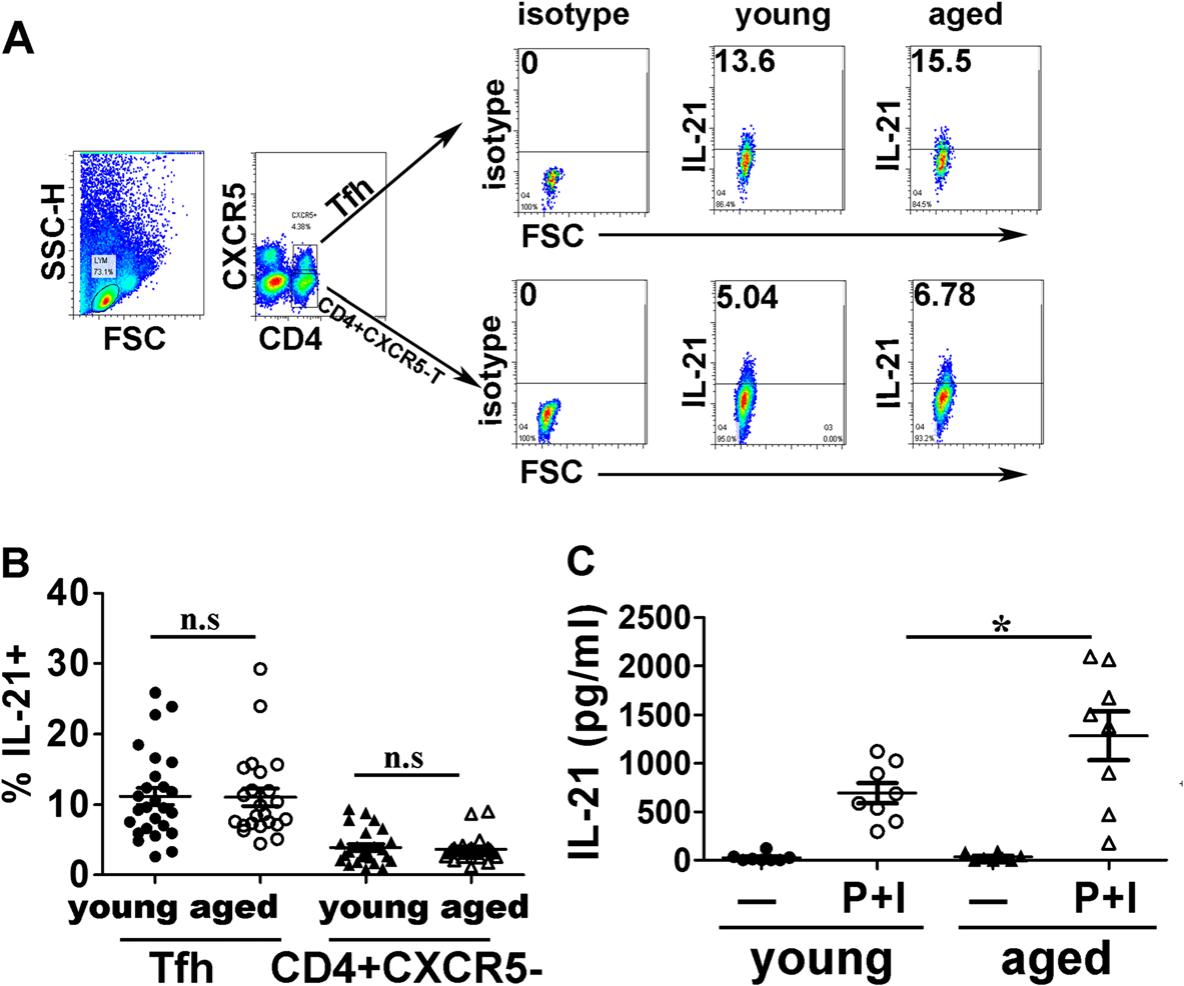
**Similar frequency of IL-21 produced by aged and young blood CXCR5 + CD4 + Tfh cells. A**. Representative results of IL-21+ cells produced by Tfh or CD4 + CXCR5-cells were shown. **B**. FACS data of percentages of IL-21+ cells in CD4 + CXCR5 + Tfh cells or CD4 + CXCR5- cells from both aged and young individuals (young subjects: n = 23, aged subjects: n = 23) were shown. **C**. IL-21 concentration determined by ELISA was shown. PBMCs were prepared and cultured with P + I (PMA and inomycin) or – (medium) for 3 days. Supernatants were collected and IL-21 was evaluated by ELISA. *: p < 0.05. All data were expressed with line at mean with SEM.

IL-21 is mainly produced by Tfh cells, and it is a very important cytokine of Tfh to help B cells. Therefore, we determined IL-21 production by CXCR5 + CD4 + Tfh or CD4 + CXCR5-T cells using intracellular cytokine staining. The results showed that both Tfh and CD4 + CXCR5-T cells could produce IL-21 (Figure 5A), and there was no significant difference of frequencies of IL-21+ cells inCD4 + CXCR5 + Tfh as well as CD4 + CXCR5-T cells from the elderly compared with young individuals (Figure 5B). Meanwhile, PBMCs were prepared from healthy aged and young individuals, and cultured in the absence or presence of PMA and inomycin for 3 days, and then the concentration of IL-21 was determined by ELISA. As shown in Figure 5C, there were very low levels of IL-21 without stimulation from both aged and young individuals. Compared with young subjects, aged PBMCs stimulation with PMA and inomycin produced significantly higher levels of IL-21. These results indicated that IL-21 production by aged PBMCs increased significantly compared with young individuals.

### Th1, Th2, Th17 and Th22 characterized cytokine production within CD4 + CXCR5 + Tfh or CD4 + CXCR5-T cells from healthy aged and young individuals

**Figure 6:**
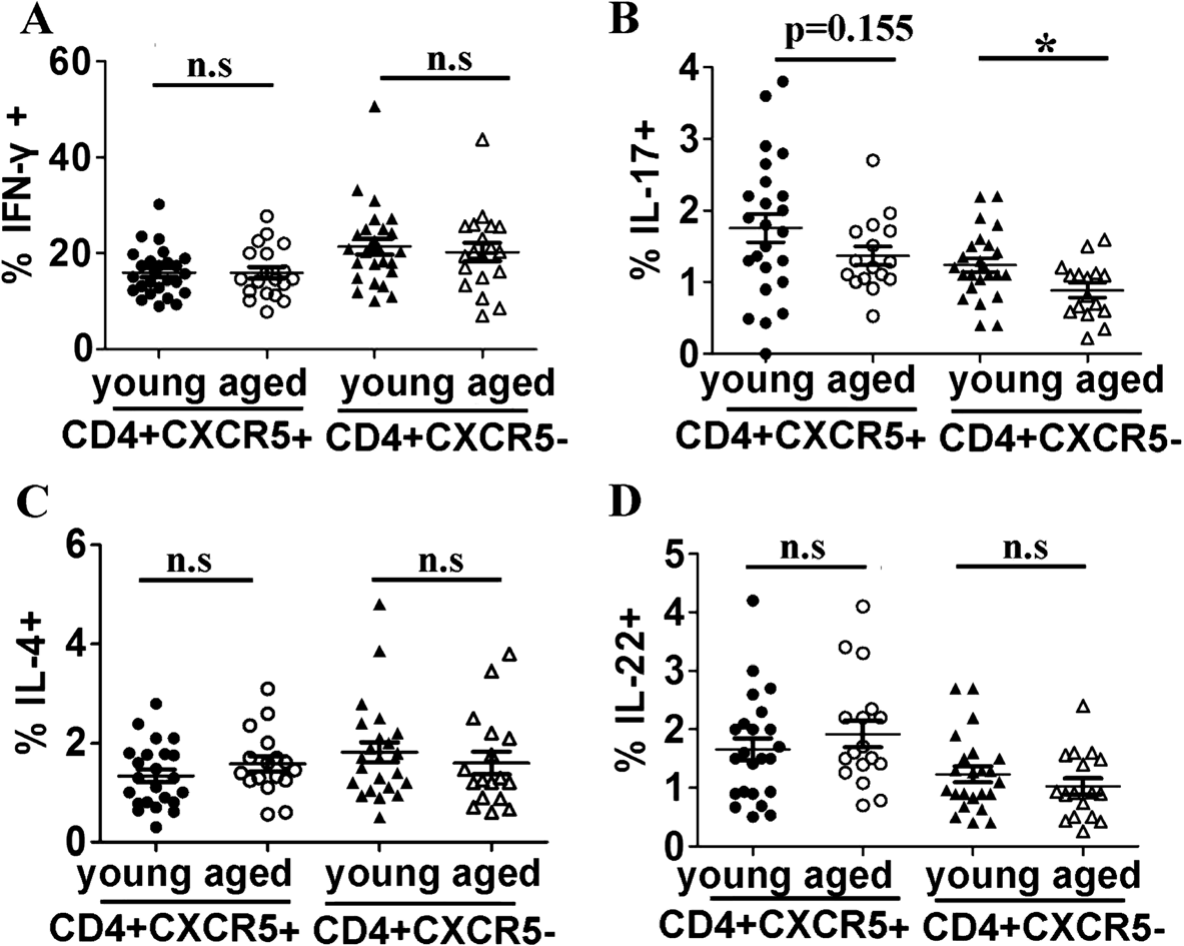
**Comparison of IFN-γ+, IL-17+, IL-4 + or IL-22+ cells among aged and young CXCR5 + CD4 + Tfh**. PBMCs were prepared, stimulated with PMA and inomycin for 4–6 hours, and intracellular staining were done. FACS data were analyzed: lymphocytes were first gated and then CD4 + CXCR5+ or CD4 + CXCR5- cells were gated and cytokines such as IFN-γ, IL-17, IL-4 and IL-22 were analyzed. Statistical data of frequencies of IFN-γ + **(A)**, IL-17 + **(B)**, IL-4 + **(C)** and IL-22 + **(D)** among CD4 + CXCR5 + Tfh or CD4 + CXCR5- cells were shown. *: p < 0.05, n.s: no significant difference. All data were expressed with line at mean with SEM.

**Figure 7:**
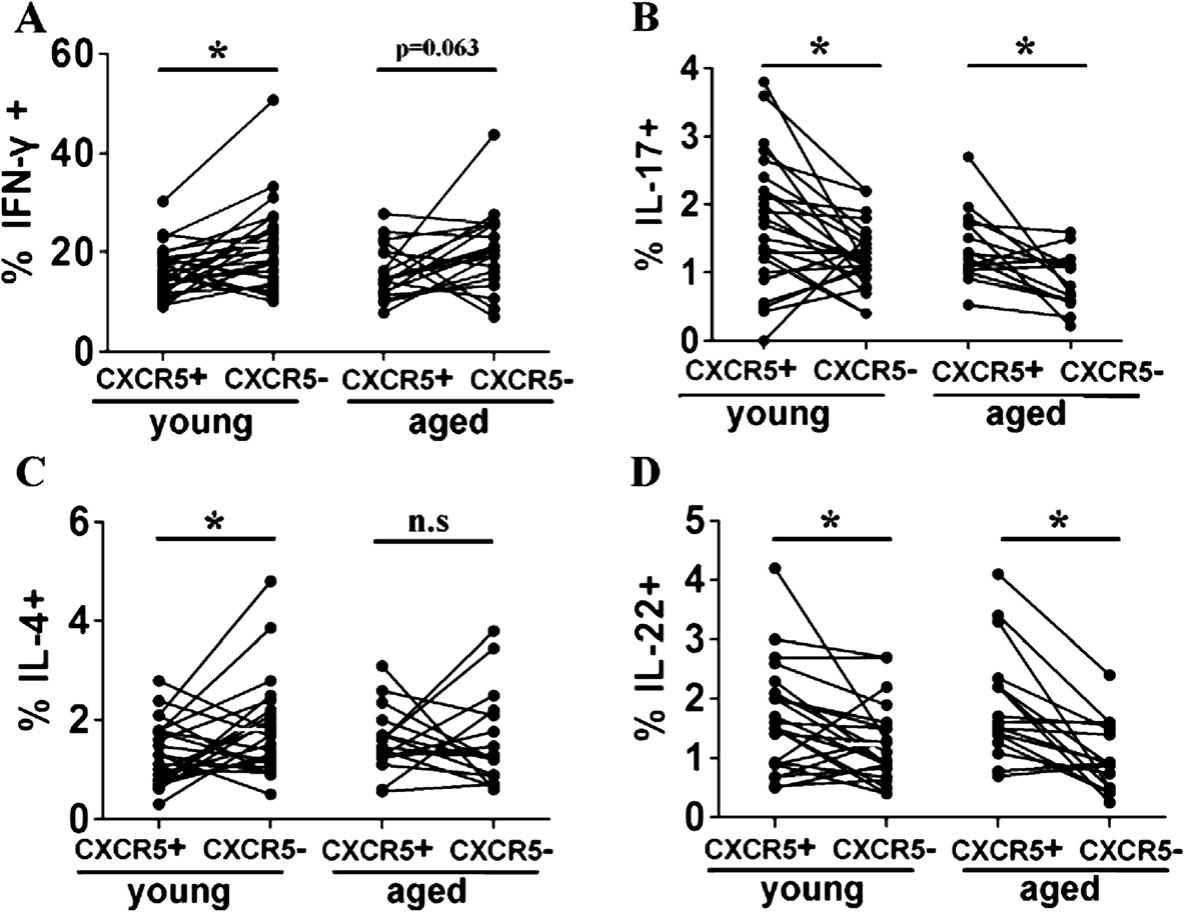
**Different frequencies of IFN-γ+, IL-17+, IL-4+ or IL-22+ cells among Tfh compared with CD4 + CXCR5- cells.** Statistical data of frequencies of IFN-γ + **(A)**, IL-17 + **(B)**, IL-4 + **(C)** and IL-22 + **(D)** among CD4 + CXCR5 + Tfh or CD4 + CXCR5- cells were shown. All data were expressed with line at mean with SEM. Differences between two groups were assessed by paired *t* test for two-tails. *: p < 0.05, n.s: no significant difference.

Finally, we evaluated Th1, Th2, Th17, and Th22 characterized cytokines IFN-γ, IL-4, IL-17 or IL-22 production within blood CD4 + CXCR5 + Tfh cells or CD4 + CXCR5-T cells from young and elderly subjects. The results showed that there were no significant differences of percentages of IFN-γ+, IL-4+, or IL-22 + cells within aged blood CD4 + CXCR5 + Tfh or CD4 + CXCR5-T cells compared with their counterparts of young individuals respectively (Figure 6A, 6C and 6D). However, frequencies of IL-17 producing cells within aged CD4 + CXCR5-T cells were markedly lower, and there was a slight tendency for IL 17-producing cells to be lower in the elderly (p = 0.155) (Figure 6B). Moreover, lower percentages of IFN-γ + or IL-4 production by young blood CXCR5 + CD4 + Tfh cells, and higher frequencies of IL-17 or IL-22 production by blood young or aged CXCR5 + CD4 + Tfh cells were observed compared with their counterparts within CD4 + CXCR5-T cells, respectively. There was no significant difference of frequencies of IFN-γ or IL-4 producing cells within aged CD4 + CXCR5-T cells and aged CXCR5 + CD4+ Tfh cells (Figure 7).

## Discussion

Tfh cells have been recently established as a novel Th subset specialized for providing help to B cells in GCs to differentiate into long-lived memory B and plasma cells [23,24]. Age-related declines in humoral responses are mainly due to defects in the cognate helper function of CD4 + T cells and in the B cells from aged individuals [15-19]. However, this phenomenon and the mechanisms of declined cognate helper activity of aged CD4 + T cells for B cells remain not well understood in human beings. In the present study, we determined whether there are alterations of Tfh cells in aging of human beings.

First, we determined peripheral blood CXCR5 + CD4 + Tfh cells from healthy young and elderly individuals. It is extremely challenging to determine GCs’ Tfh cells through obtaining lymph node samples from healthy humans routinely. As demonstrated by one study published on immunity, human blood CXCR5 + CD4 + T cells shared functional properties with Tfh cells and appeared to represent their circulating memory compartment [28]. Therefore, we defined blood CXCR5 + CD4 + T cells as peripheral blood Tfh by FACS. The results demonstrated that frequencies of aged CXCR5 + CD4 + Tfh cells were higher than those of young Tfh cells, and correlated with age (Figure 1). Consistent with our results, aged mice have increased numbers of isolated lymphoid follicles (ILFs), an increased T-lymphocyte population within aged ILFs [29]. We observed that there were no significant differences of percentages or cell counts of CD4 + T cells. Different literatures reported different data about change of CD4 + T number and percentage in the elderly. Hirokawa et al. observed an age-related increase in the number of CD4+ T cells in the Japanese population [30]. Provinciali et al’s results showed the absolute number of total CD4+ T cells was progressively reduced with increasing age in Italian population [31]. Lower cell counts not percentages of CD4 + T cells in the elderly in Hongkong were observed [32]. The different data might be due to race, sex, CMV infection, and other factors impact on subpopulation of CD4 + T cells [32,33].

Then, we evaluated and compared the memory phenotype CD45RO and CCR7and co-stimulatory molecule CD28 expression on CXCR5 + CD4 + Tfh cells. The results showed that both aged and young blood CXCR5 + CD4 + Tfh cells constitutively expressed CD45RO and CCR7, and there was no difference between young and aged subjects (Figures 2 and 3). As we know, both CD45RO and CCR7 are expressed by central memory T cells [34]. Consistent with previous reports [28,35-37], blood CXCR5 + CD4 + Tfh cells co-expressing CD45RO and CCR7 belong to memory T cells. In addition, similar with other studies, we observed that aged memory CD4+ T cells accumulated increased [10,11,38]. Moreover, we demonstrated that blood CXCR5 + CD4 + Tfh cells constitutively expressed co-stimulatory molecule CD28 (Figures 2 and 3). CD28, a major co-stimulatory receptor, was responsible for the optimal antigen-mediated T-cell activation, proliferation and survival of T cells. Previous reports demonstrated that CD28 was essential for Tfh development [39]. Furthermore, decreased CD28 expression on aged CD4 + T, CD3 + CD4-T or CXCR5-CD4 + T cells was observed in this study. Consistent with our results, the accumulation of CD28 (-) T cells, particularly within the CD8 subset, is one of the most prominent changes during in the age-associated decline of immune function in humans [40]. Blood CXCR5 + CD4 + Tfh cells constitutively expressed CD28, which indicated that these cells could be fully activated and play a key role in development and function of CXCR5 + CD4 + Tfh cells from both aged and young subjects.

IL-21 is the most potent cytokine known for driving B cells differentiation into plasma cells in both mice and humans, and is highly secreted by Tfh cells, although not exclusively secreted by Tfh cells [27,41,42]. In this study, we demonstrated aged PBMCs produced significantly higher levels of IL-21compared with young subjects (Figure 5). Consistent with our observation, there has been recently reported that aged CD4 + T cells from healthy elderly individuals secreted significantly higher levels of IL-21 on priming with dendritic cells (DC) under anti-CD3 plus anti-CD28 mAbs [43], and IL-21 was mainly produced by memory CD4 + T cells [44]. There were higher memory CD4 + T cells in aged subjects, therefore there were higher levels of IL-21 produced by aged PBMCs. In contrast, there has been reported that aged memory CD4 + T cells expressed lower levels of IL-21 after stimulated with anti-CD3 plus anti-CD28 evaluated by real time RT-PCR [45]. The different results might be due to different experimental condition. IL-21 is a cytokine that has broad effects on immune and non-immune cells, and more and more data suggest this cytokine is critically involved in the initiation and/or progression of inflammatory reactions where self-reactive immune cells or antibodies cause damage in tissue [46]. Increased IL-21 production by aged PBMCs may significantly impact immune functions in elderly individuals, and contribute to aging associated inflammatory condition or diseases.

Recently, there was one literature which reported that human blood CXCR5 + CD4 + Tfh cells comprised three subsets: Th1, Th2 and Th17 cells with different capacity to help B cells. Th2 and Th17 cells within CXCR5+, but not within CXCR5-, compartment efficiently induced naïve B cells to produce Abs. In contrast, Th1 cells from both CXCR5+ and CXCR5- compartments lacked the capacity to help B cells [28]. Our results demonstrated that there were no significant differences of percentages of IFN-γ, or IL-4 production by aged blood CD4 + CXCR5 + Tfh or CD4 + CXCR5-T cells compared with their counterparts of young individuals (Figure 6). Previous studies about age-associated change in IFN-γ or IL-4 production by T cells were largely inconsistent [47]. The frequencies of IL-17+ cells produced by aged CD4 + CXCR5-T cells were markedly lower, and frequencies of IL-17 production by aged Tfh cells didn’t decrease significantly compared with young subjects (Figure 6B).Consistent with our results, previous study demonstrated that purified memory CD4 + T cells from aged people produced lower levels of frequency of Th17 cells compared with the young [48].

## Conclusions

In conclusion, in the present study we observed that frequencies of aged blood CXCR5 + CD4 + Tfh cells increased. Aged or young blood CXCR5 + CD4 + Tfh cells constitutively expressed CD28, and were resting T memory cells. There were similar frequencies of Th characterized cytokine production such as IL-21, IFN-γ, IL-4, IL-17 or IL-22 by aged and young Tfh cells. However, aged PBMCs produced significantly higher amount of IL-21. Future studies should observe whether there are defects of aged Tfh cells in helping B cells to produce Abs.

## Materials and methods

### Ethics statement

This study was conducted in compliance with the Declaration of Helsinki and applicable national laws and regulations, and was approved by the ethics committee of Guangzhou Medical University (Guangzhou, China) and the ethics committee of Guangdong General Hospital, Guangdong Academy of Medical Sciences (Guangzhou, China). And written informed consent was obtained from all healthy aged and young donors or the parents of young children who participated in this study.

### Subjects

Healthy young (age range: 22–39 years old, average age: 29.5, male: n = 25, female: n = 20) and elderly (age range: 60–86 years old, averaged age: 71.0, male: n = 19, female: n = 24,) volunteers were critically selected on the basis of clinical records and laboratory examinations from Guangdong General Hospital. Healthy young children (age: 0.8-2.8 years old, average age: 2.1 years old, 4 girls, 4 boys) were recruited from Guangdong General Hospital. We regarded subjects as being healthy if they had no neoplastic or autoimmune diseases, EBV, HBV, HCV, TB infections, or serious prior illnesses, were receiving on no medication other than anti-hypertensive medication. Routine laboratory examinations of the serum of these individuals were performed to examine the liver and kidney functions. We did not investigate whether the subjects had previous infection with cytomegalovirus (CMV) (Prevalence of CMV infection is 90-100% in developing countries) [49].

### Reagents

Anti-CD4 PerCP, anti-CD4 PerCP-cy5.5, anti-CD3 APC, anti-CXCR5-Alexafluor 488, anti-CD45RO PE, anti-CCR7 PE, anti-CD28 PE, anti-CD69 PE, anti-HLA-DR PE, anti-IL-21 PE, anti-IL-4 PE, anti-IFN-γ APC and isotype-matched control mAbs were purchased from BD PharMingen (San Diego, CA, USA). Anti-IL-17 APC and ELISA for determining IL-21 was purchased from eBioscience (San Diego, CA, USA). Anti-IL-22 PE was purchased from R & D Systems (Abingdon, UK). PMA, ionomycin (INO), saponin and Brefeldin A were purchased from Sigma-Aldrich (Fluka, Sigma, USA).

### Cell surface staining

Heparinized blood was collected, then 50 ul blood per tube was added, and anti-CD4 PerCP, anti-CD3 APC, anti-CXCR5-Alexafluor488, anti-CD45RO PE, anti-CCR7 PE, anti-CD28 PE, anti-CD69 PE, anti-HLA-DR PE, and isotype-matched control mAbs were added into tube and mixed, incubated 15–20 minutes at room temperature. Erythrocytes were lysed with ammonium chloride (NH_4_Cl) for 5–8 minutes. Cells were washed, re-suspended with staining buffer, and detected by FACS Calibur (BD Biosciences, San Jose, CA). The data were analyzed using FlowJo software (Tree Star, Ashland, OR, USA). Leukocyte count and differential were determined with a routine hematology analyzer (COULTER LH780, Beckman Coulter Inc., USA). The absolute counts of each lymphocyte subpopulation were calculated by multiplying the relative size of the lymphocyte subpopulation and the absolute lymphocyte count.

### PBMCs preparation

Peripheral blood mononuclear cells (PBMCs) were isolated from heparinized blood of healthy donors using Ficoll-Hypaque density gradient centrifugation, and washed twice in Hank’s balanced salt solution. These cells were finally adjusted to a final concentration of 2 × 10^6^ /ml in complete RPMI 1640 medium (GIBCO, Grand Island, NY, USA) supplemented with 10% FCS (Sijiqing, China), 100 U/mL penicillin, 100 mg/mL streptomycin, 50 mM 2-mercaptoethanol, and 2 mM L-glutamine (all from GIBCO).

### Intracellular cytokine staining

PBMCs were stimulated with PMA (20 ng/ml) and ionomycin (1ug/ml) for 4–6 hours at 37°C in a 5% CO_2_ humidified atmosphere. At the end of first hour during the incubation, brefeldin A (BFA, 10 μg/ml) was added into the culture. The cells were collected, washed twice with cold PBS, and cells surface staining ofanti-CD4 PerCP-cy5.5, anti-CXCR5-Alexafluor 488 was added for 20-30 min. Cells were washed with PBS, fixed with 4% paraformaldehyde and re-suspended in permeabilization buffer (PBS containing 0.1% saponin and 0.5% BSA). After incubation at 4°C for 2 hours or overnight, intracellular cytokine staining of Anti-IL-21 PE, Anti-IL-22 PE, Anti-IL-4 PE, Anti-IL-17 APC, anti-IFN-γ APC, and isotype-matched control mAbs were added and incubated at 4°C for 25–30 min. Cells were washed with PBS, resuspended in cold staining buffer, and detected by FACSCalibur (BD Biosciences, San Jose, CA). The data were analyzed using FlowJo software (Tree Star, Ashland, OR, USA).

### Cell culture and ELISA for IL-21

PBMCs were prepared, adjusted to a final concentration of 2 × 10^6^ /ml, stimulated with PMA and inomycin or medium, and cultured for 3 days. Cell free culture supernatants were harvested and IL-21 was quantified by enzyme-linked immunosorbent assay (ELISA) according to the manufacturer’s instruction. The sensitive of ELISA kit was 31 pg/ml for IL-21.

### Statistical analysis

Comparison between two groups was assessed by Mann Whitney test for two-tails (except Figure 7, performed by paired *t* test for two tails). P value of < 0.05 was considered to be statistically significant. Data were expressed with mean with SEM in results. All statistical analyses were performed using GraphPad Prism (version 5.0 Software Inc, San Diego, CA, USA).

## Competing interests

The authors declare that they have no competing interests.

## Authors’ contributions

Conceived and designed the experiments: ES MZ. Performed the experiments: MZ RZ HG ZL FL. Analyzed the data: MZ TL YL. Contributed reagents/materials/analysis tools: ES MZ XC FH. Wrote the paper: ES RZ. All authors read and approved the final manuscript.
