# Erratum to: The effect of aging on the frequency, phenotype and cytokine production of human blood CD4 + CXCR5 + T follicular helper cells: comparison of aged and young subjects

**DOI:** 10.1186/s12979-015-0039-7

**Published:** 2015-09-29

**Authors:** Maohua Zhou, Ruqiong Zou, Huiquan Gan, Zhimei Liang, Fujun Li, Ting Lin, Yanfei Luo, Xiaoming Cai, Fang He, Erxia Shen

**Affiliations:** Department of Pathogenic Biology and Immunology, Guangzhou Hoffmann Institute of Immunology, School of Basic Sciences, Guangzhou Medical University, Guangzhou, 510182 China; Department of Pathology and Laboratory Medicine, Guangdong General Hospital, Guangdong Academy of Medical Sciences, Guangzhou, 510080 China; Department of Obstetrics and Gynecology, The Third Affiliated Hospital of Guangzhou Medical University, Guangzhou, 510150 China

## Erratum

After publication of this work [1], we noted that this article’s first address should have been stated as Guangzhou Medical University. We have changed it accordingly without changing the authors’ order.
